# ALX/FPR2 Modulates Anti-Inflammatory Responses in Mouse Submandibular Gland

**DOI:** 10.1038/srep24244

**Published:** 2016-04-11

**Authors:** Ching-Shuen Wang, Yinshen Wee, Chieh-Hsiang Yang, James E. Melvin, Olga J. Baker

**Affiliations:** 1 School of Dentistry, University of Utah, Salt Lake City, UT 84108, USA; 2 The Division of Microbiology and Immunology, Department of Pathology, University of Utah School of Medicine, Salt Lake City, UT, 84102, USA; 3 Department of Bioengineering, University of Utah, Salt Lake City, Utah, 84112, USA; 4 National Institute of Dental & Craniofacial Research, NIH, Bethesda, MD 20892, USA.

## Abstract

Activation of the G-protein coupled formyl peptide receptor 2 (ALX/FPR2) by the lipid mediators lipoxin A_4_ and resolvin D1 (RvD1) promotes resolution of inflammation. Our previous *in vitro* studies indicate that RvD1 activation of ALX/FPR2 resolves cytokine-mediated inflammatory responses in mammalian cells. However, the impact of ALX/FPR2 activation on salivary gland function *in vivo* is unknown. The objective of this study was to determine whether submandibular glands (SMG) from *ALX*/*FPR2*^*−*/*−*^ mice display enhanced inflammatory responses to lipopolysaccharides (LPS) stimulation. For these studies, C57BL/6 and *ALX/FPR2*^−/−^ mice at age 8-12-week-old were treated with LPS by *i.p* for 24 h. Salivary gland structure and function were analyzed by histopathological assessment, saliva flow rate, quantitative PCR, Western blot analyses and immunofluorescence. Our results showed the following events in the *ALX/FPR2*^−/−^ mice treated with LPS: a) upregulated inflammatory cytokines and decreased M3R (Muscarinic Acetylcholine receptor M3) and AQP5 (Aquaporin 5) protein expression, b) decreased saliva secretion, c) increased apoptosis, d) alteration of tight junction and neuronal damage. Overall, our data suggest that the loss of ALX/FPR2 results in unresolved acute inflammation and SMG dysfunction (xerostomia) in response to LPS that is similar to human salivary gland dysfunction induced by bacterial infection.

Acute salivary gland bacterial infection occurs frequently in patients infected by both gram-positive (*e.g. Streptococcus pneumoniae*) and gram-negative bacteria (*e.g. Escherichia coli*)^1^,^2^. In most cases, the infection leads to increased inflammation and pain of the major salivary glands^3^. Additionally, patients with salivary gland infection experience xerostomia (dry-mouth), which significantly impacts quality of life if left unresolved^4^. Several case-report studies found that infection of the major salivary glands primarily occurs in susceptible elderly and newborn populations^5^,^6^. For example, acute sialadenitis (AS) usually occurred by bacterial infection in salivary glands, leading to salivary gland dysfunction^2^. The incidence of AS has been documented in 0.01 to 0.02% of total hospital admissions internationally^6^,^7^. Submandibular gland (SMG) infection is estimated to be present in approximately 10% of all AS cases^7^. To treat such disease, high doses of antibiotics are often applied that frequently lead to drug-resistance. Besides bacterial infection, systemic infection induced by viruses (*e.g.* Herpes virus and Hepatitis C virus) will also cause SMG dysfunction, leading to xerostomia^1^. The exact mechanism of AS is unclear; however, unresolved inflammatory responses may play a role in this condition^9^,^10^.

Resolution of acute inflammation is vital in returning salivary glands to homeostasis while impeding chronic inflammation. Chronic inflammation of the salivary glands, as occurs in Sjögren’s Syndrome (SS), leads to salivary gland dysfunction^11^,^12^. There is no immediate cure for SS. Thus, novel animal models with enhanced inflammation may provide new insights for understanding salivary gland dysfunction under inflammatory conditions.

Mammalian cells convert ω-3 poly unsaturated fatty acids into resolvins (Rvs), which are highly potent anti-inflammatory compounds that regulate the inflammation cascades in models of complex diseases^8^,^13^,^14^,^15^,^16^,^17^,^18^,^19^. Resolvin D1 (RvD1, 7*S*, 8*R*, 17*S* trihydroxy docosahexaenoic acid [DHA]) is produced by an interaction between leukocytes and endothelial or epithelial cells^20^. Our recent studies have highlighted the role of RvD1 and its receptor ALX/FPR2 in the resolution of salivary gland inflammation^14^,^21^. Specifically, we have shown that ALX/FPR2 is expressed and functional in the rat parotid cell line Par-C10 and in mouse SMG^21^. Furthermore, RvD1 activation of the ALX/FPR2 prevents TNF-α-mediated inflammation in Par-C10, leading to activation of survival pathways and improving epithelial integrity^14^. RvD1 activation also reduces TNF-α-mediated inflammation in mouse SMG by blocking caspase-3 activation, which triggers phosphorylation of Erk1/2 and Akt to improve cell survival^21^. Finally, we demonstrated that the machinery to produce RvD1 is expressed in mouse and human salivary glands^22^. However, the *in vivo* role of ALX/FPR2 in modulating mouse SMG inflammation has not been investigated.

The goal of this study was to determine whether SMG from *ALX/FPR2*^−/−^ mice display enhanced inflammatory responses to lipopolysaccharides (LPS) stimulation, and thus, provide a pre-clinical model to test anti-inflammatory therapeutic interventions.

## Results and Discussion

### *ALX/FPR2* ^−/−^ mice treated with LPS showed no difference in leukocyte infiltration in SMG

**Figure 1:**
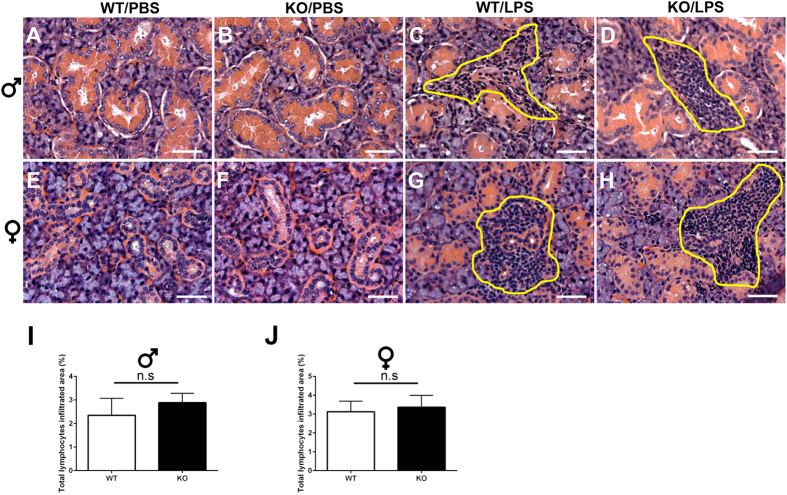
*ALX/FPR2*^−/−^ and C57BL/6 mice treated with LPS showed similar inflammatory infiltration in SMG. Representative SMG sections from male (**A–D**) and female (**E–H**) mice treated with either PBS or LPS. Abbreviations: *WT/PBS* wild type C57BL/6 mice injected with PBS, *KO*/*PBS ALX/FPR2*^−/−^ mice injected with PBS, *WT*/*LPS* wild type C57BL/6 mice injected with LPS, and *KO*/*LPS ALX/FPR2*^−/−^ mice injected with LPS. Lymphocytic infiltrations are circled in yellow. Scale bars = 100 μm. The percentage of infiltrated lymphocytes per each tissue section of male (**I**) and female (**J**) was calculated as described in Methods. Data represent the mean ± SD from six mice per group. n.s., no significance.

SMG from different treatment groups were analyzed by H&E histological staining. First, we compared SMG from the PBS injected C57BL/6 and *ALX/FPR2*^−/−^ mice, and found that both animals showed healthy SMG (*i.e*., no developmental abnormalities or changes in the SMG phenotype, Fig. 1A,B,E,F). These results are consistent with previous reports indicating that *ALX/FPR2*^−/−^ mice do not display developmental abnormalities^23^. However, this is the first time SMG have been studied in this model.

When C57BL/6 or *ALX/FPR2*^−/−^ mice were injected with LPS, we observed that they developed SMG immune cell infiltration at 24 h (Fig. 1C,D,G,H). However, we did not observe a qualitative gross difference between C57BL/6 and *ALX/FPR2*^−/−^ mice in terms of SMG inflammation (circled region in Fig. 1C,D,G,H). Further quantification of the area infiltrated with lymphocytes in the whole SMG showed no statistical significance between C57BL/6 and *ALX/FPR2*^−/−^ mice treated with LPS (Fig. 1I,J). We found that both genders showed similar SMG inflammation responses to either PBS or LPS stimulation. These results suggest no gender differences in terms of SMG inducible inflammatory events.

Although SMG from *ALX/FPR2*^−/−^ mice treated with LPS did not show a difference in the number of immune cells as compared with C57BL/6 mice (Fig. 1), it was important to accurately confirm the degree of infiltrating immune cells into the SMG. Therefore, we performed FACs by staining SMG cells from homogenates with the leukocyte marker CD45. Our results showed that *ALX/FPR2*^−/−^ mice treated with LPS display a trend towards a lower number of leukocytes (CD45^+^) in SMG from both genders (Supplementary Fig. 2). However, these results are not statistically significant, but they are consistent with the histopathological analysis, further reinforcing that there are no differences in immune cell infiltration between the groups studied. It will be interesting to further analyze the subpopulation of these infiltrating immune cells in SMG in the future.

We did not examine other organs to determine the degree of immune cell infiltrations in *ALX/FPR2*^−/−^ mice treated with LPS. However, previous reports in other disease models have shown that the degree of immune cell infiltration is organ-specific and dependent on time course of infection and type of pathogens^24^,^25^,^26^. Under these conditions, it is clear that ALX/FPR2 deficiency leads to inappropriate neutrophil apoptosis and alteration of the macrophage populations (*i.e.,* polarization M1 or M2 subtypes)^8^,^27^,^28^, resulting in disease progression in several inflammatory models^23^,^24^,^25^,^26^,^28^,^29^,^30^,^31^ including the present study. Together, these results further emphasize the critical role of ALX/FPR2 in regulating acute tissue inflammation and resolution.

### *ALX/FPR2* ^−/−^ mice treated with LPS showed increased expression of SMG pro-inflammatory cytokines

**Figure 2:**
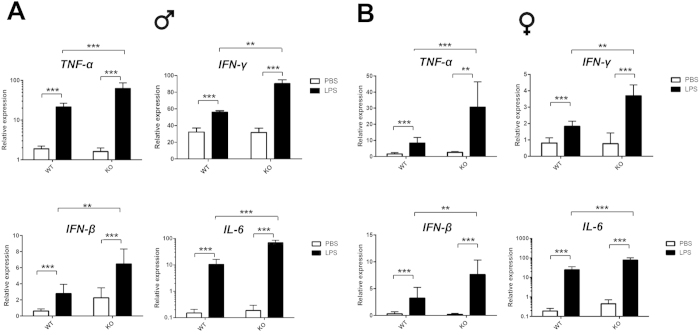
*ALX/FPR2*^−/−^ mice treated with LPS showed increased SMG pro-inflammatory cytokines expression. qPCR analysis of cytokine expression levels from SMG with PBS (white bars) and LPS (black bars) treatments. Cytokine expression in male (**A**) and female (**B**) groups. Abbreviations: *WT* wild type C57BL/6 mice, *KO ALX/FPR2*^−/−^ mice. N = 6 mice were used for each experimental group. Data are given as mean ± SD; ** and ***, *P* < 0.01 and *P* < 0.001 respectively.

To determine inflammatory cytokine profiles in SMG from C57BL/6 and *ALX/FPR2*^−/−^ mice treated with PBS or LPS, we performed qPCR analysis. Our results indicated that the pro-inflammatory cytokines TNF-α, IL-6, IFN-β and IFN-γ were significantly upregulated in *ALX*/*FPR2*^−/−^ mice treated with LPS as compared to C57BL/6 mice, while no upregulation of pro-inflammatory cytokines was observed in animals treated with PBS (Fig. 2). These results are consistent with a previous study indicating that *ALX/FPR2*^−/−^ mice stimulated by LPS injection showed an enhanced acute inflammatory response in a liver injury model^25^. Upregulation of pro-inflammatory cytokines has been observed in SS and AS^1^,^2^,^12^,^35^,^36^. Our previous work showed that ALX/FPR2 activation with RvD1 prevents TNF-α-mediated inflammation in Par-C10 cells and activates survival pathways thus improving epithelial integrity^14^. Furthermore, RvD1 reduces TNF-α-mediated inflammation in freshly isolated mouse SMG by blocking caspase-3 activation, which triggers phosphorylation of Erk1/2 and Akt to improve cell survival^21^. Together, these studies indicate that RvD1 affects downstream signaling events induced by TNF-α. Consequently, in this *in vivo* study, ALX/FPR2 deficiency might increase both TNF-α production and intracellular responses of salivary gland epithelial cells to this cytokine (Fig. 2A,B). These events are likely to influence the outcome caused by ALX/FPR2 deficiency and indicate that the ALX/FPR2 has a protective role in SMGs during inflammation associated with the acute phase response.

### *ALX/FPR2* ^−/−^ mice treated with LPS showed impaired saliva secretion

**Figure 3:**
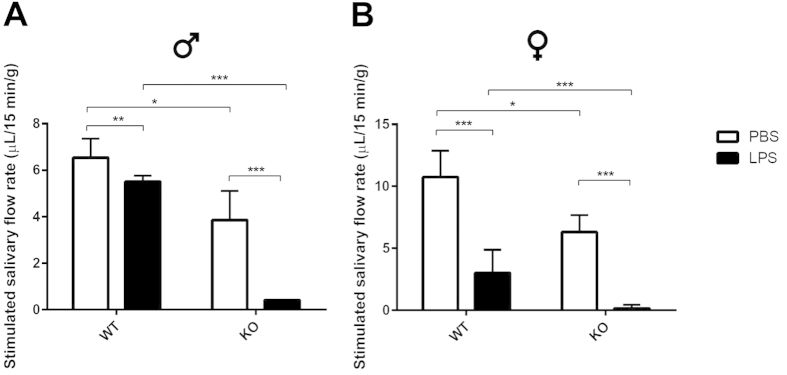
*ALX/FPR2*^−/−^ mice treated with LPS showed impaired saliva secretion. Saliva flow rate of male (**A**) and female (**B**) mice treated with PBS (white bars) and LPS (black bars) treatments. Abbreviations: *WT* wild type C57BL/6 mice, *KO ALX/FPR2*^−/−^ mice. N = 6 mice were used per condition. Data are given as mean ± SD; *, ** and ***, *P* < *0.05, P* < 0.01 and *P* < 0.001 respectively.

Given that *ALX/FPR2*^−/−^ mice showed persistent inflammation in response to LPS, we predicted that salivary gland function was affected. To test this hypothesis, we measured the flow rate of pilocarpine-stimulated saliva secretion. Our results indicate that saliva secretion was almost completely abolished in *ALX/FPR2*^−/−^ mice from the LPS stimulation group (Fig. 3). Similar to what we observed in the histopathological studies, we found no gender differences between the groups studied here. Somewhat surprisingly, our results show that saliva secretion was also significantly reduced in both genders of *ALX/FPR2*^−/−^ mice with PBS treatment (Fig. 3). Previous studies indicated that salivary gland dysfunction can be caused by several factors such as inflammatory cellular responses and alterations in tight junction integrity. Particularly, activation of ALX/FPR2 is critical in regulating tight junction integrity in several models of inflammation (*e.g*., salivary gland epithelium, human airway epithelium and blood brain barrier)^14^,^38^,^39^. These results demonstrate that ALX/FPR2 is a critical player in regulating inflammation and maintaining tight junction integrity.

Taken together, our functional results share some clinical features with human AS or SS. Specifically, the condition is caused by *S. aureus* and is characterized by acute pain, swelling and xerostomia and commonly observed in hospitalized patients^2^,^6^,^40^. It would be tempting to speculate that patients with AS or SS may have functional alteration of the ALX/FPR2; however, future studies are needed to confirm this notion.

### LPS treatment down-regulated M3R and AQP5 expression in SMG of *ALX/FPR2* ^−/−^ mice

**Figure 4:**
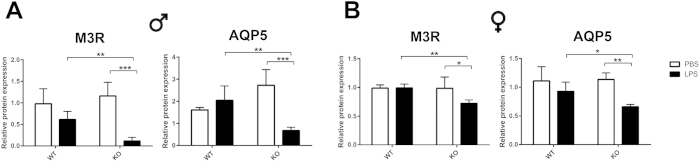
*ALX/FPR2*^−/−^ mice treated with LPS displayed M3R and AQP5 down-regulation in SMG. M3R and AQP5 protein expression levels in male (**A**) and female (**B**) groups. Abbreviations: *WT* wild type C57BL/6 mice, *KO ALX/FPR2*^−/−^ mice. *PBS* mice injected with PBS, *LPS* mice injected with LPS. N = 6 mice were used for each experimental group. Data are given as mean ± SD; *, ** and ***, *P* < 0.05, *P* < 0.01 and *P* < 0.001 respectively.

To determine the possible causes of LPS-induced salivary secretory dysfunction we studied the expression of the M3R and the AQP5 water channel, both of which have been shown to major play a role in saliva secretion^41^,^42^. We found that the M3R and AQP5 protein levels were significantly down-regulated in both male and female *ALX/FPR2*^−/−^ mice treated with LPS as compared to PBS treated mice, but not in C57BL/6 mice (Fig. 4A,B). These results are consistent with previous studies showing that a decrease of M3R or AQP5 causes loss of saliva secretion in xerostomia or SS^43^,^44^. Overall, we conclude that two key players in regulating saliva secretion, M3R and AQP5, were affected in *ALX/FPR2*^−/−^ mice after 24 h stimulation with LPS.

### *ALX/FPR2* ^−/−^ mice treated with LPS showed increased apoptosis in SMG

**Figure 5:**
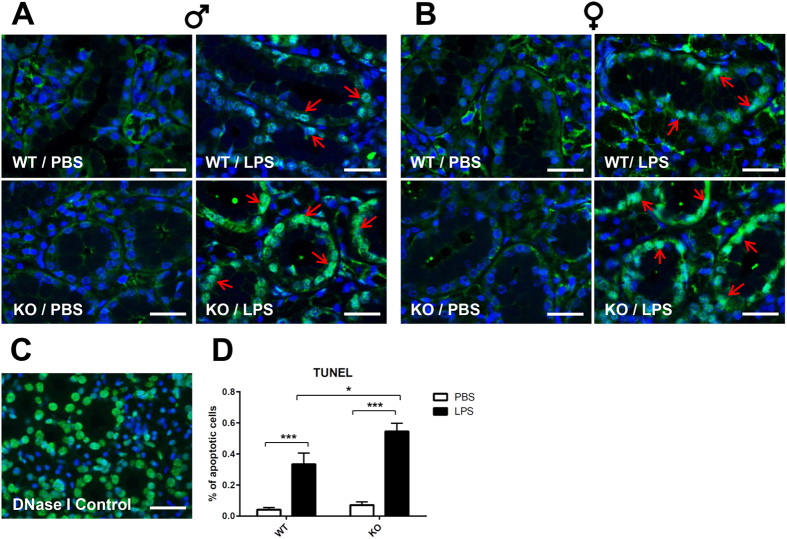
*ALX/FPR2*^−/−^ mice treated with LPS showed increased apoptosis in SMG. Representative fluorescent images of TUNEL analyses from male (**A**) and female (**B**) groups. DNase I digested experimental control is shown (**C**). Statistical analysis showed percentage of apoptotic cells (TUNEL-positive green cells/total cells stained with DAPI in blue color) from each group (n = 20 images of each group, **D**). Red arrows indicated TUNEL-positive cells. Data are given as mean ± SD; * and ***, *P* < 0.05 and *P* < 0.001 respectively. Abbreviations: *WT*/*PBS* wild type C57BL/6 mice injected with PBS, *KO*/*PBS ALX/FPR2*^−/−^ mice injected with PBS, *WT*/*LPS* wild type C57BL/6 mice injected with LPS, and *KO*/*LPS ALX/FPR2*^−/−^ mice injected with LPS. N = 6 mice were used for each experimental group. Scale bars in all panels =50 μm.

The M3R and AQP5 protein expression levels did not directly correlate with the nearly total loss of saliva secretion observed in *ALX/FPR2*^−/−^ mice treated LPS (as we still see expression of these proteins in the *ALX/FPR2*^−/−^ mice, Fig. 3). Therefore, we investigated whether LPS caused salivary gland cell death, thus leading to a dramatic >90% loss of saliva secretion in SMG. We performed a TUNEL assay to monitor apoptosis. Our results showed significantly higher fluorescent signals in SMG tissue sections from both male and female *ALX/FPR2*^−/−^ mice treated with LPS as compared with C57BL/6 mice (Fig. 5). The fluorescent signals were observed in both acinar and ductal cells, consistent with SMG apoptosis in *ALX/FPR2*^−/−^ mice (Fig. 5A,B). Our results are consistent with our previous studies indicating that LPS causes apoptosis in salivary cells *in vivo*^32^,^33^,^34^. However, this is the first study to show that absence of ALX/FPR2 leads to an exacerbated response to LPS.

### *ALX/FPR2* ^−/−^ mice treated with LPS showed altered expression and localization of β-III tubulin and tight junction protein ZO-1 in SMG

**Figure 6:**
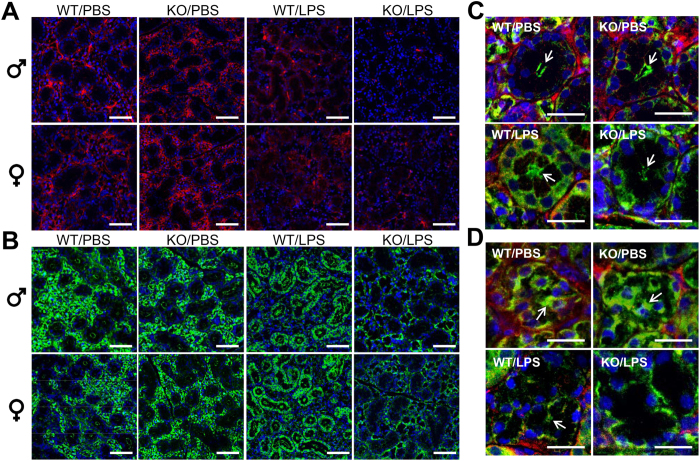
*ALX/FPR2*^−/−^ mice treated with LPS showed altered expression and localization of beta tubulin III and ZO-1 in SMG. Confocal images were total projected in x-y planes (z = 7 μm) from each treatment group with 10× objective. Anti-β tubulin III (red) staining is shown in (**A**) and anti-ZO-1 (green) is shown in (**B**) of each treatment from male and female groups. Overlay images of β tubulin III (red) and ZO-1 (green) in higher magnification (20× objective) of mucous acini structure from different group of male mice are shown in (**C**). Overlay images of β tubulin III (red) and ZO-1 (green) in higher magnification (20× objective) of serous acini structure from different group of male mice are shown in (**D**). TO-PRO-3 iodide DNA staining is shown in blue. Abbreviations: *WT*/*PBS* wild type C57BL/6 mice injected with PBS, *KO*/*PBS ALX*/*FPR2*^−/−^ mice injected with PBS, *WT*/*LPS* wild type C57BL/6 mice injected with LPS, and *KO*/*LPS ALX/FPR2*^−/−^ mice injected with LPS. N = 6 mice were used for each experimental group. Scale bars in (**A,B**) =100 μm, in (**C,D**) =50 μm.

It is well known that salivary fluid secretion is controlled by parasympathetic nerves that activate acinar cell M3R to increase the intracellular [Ca^2+^] and subsequently to stimulate saliva secretion^47^,^48^. Therefore, we hypothesized that the neurons that innervate the SMG could also be affected by the LPS-induced inflammation, thus affecting nerve morphology. In order to verify this notion, we determined the expression of the neuron specific marker β-III tubulin. We visualized neuronal morphology using confocal microscopy and obtained the views from total projections of the x-y plane from 7 μm samples, which gave consistent results in both genders (Fig. 6A). Our results indicate that mice injected with PBS showed intact neuron bodies and axons in all planes (Fig. 6A). In contrast, C57BL/6 mice challenged with LPS showed a punctate staining of β-III tubulin (Fig. 6A). Moreover, there was a significant loss of fluorescent intensity in *ALX/FPR2*^−/−^ mice treated with LPS, consistent with a loss of neurons in SMG (Fig. 6A). Previous studies demonstrated that LPS triggers systematic inflammation leading to either central or peripheral nerve damage in other disease models^49^,^50^,^51^. We conclude that not only acinar and ductal cells were damaged, but the local neurons that innervate SMG were affected by LPS treatment in *ALX/FPR2*^−/−^ mice due to unresolved inflammation.

Moreover, we found the localization and expression of tight junction protein ZO-1 was also altered in *ALX/FPR2*^−/−^ mice treated with LPS of both genders (Fig. 6B). Therefore, the representative results of male groups are shown with higher magnification in Fig. 6C,D. In C57BL/6 and *ALX/FPR2*^−/−^ mice injected with PBS, we found that the apical localization and expression of ZO-1 was not affected in mucous (Fig. 6C as indicated in white arrows) and serous acini (Fig. 6D as indicated in white arrows). However, we still observed a decrease in saliva secretion in this group, indicating that ZO-1 function may be altered during this process (Fig. 3). Previous studies showed that tight junction structure and function are altered during inflammation^37^,^52^ and are regulated by ALX/FPR2 activity^14^,^38^. A limitation of this study is that other tight junction proteins such as claudins, occludin and junctional adhesion molecules were not investigated. It will be important to understand the expression and distribution of these molecules in SMG from animals treated with or without LPS in future studies.

Interestingly, C57BL/6 mice treated with LPS showed both basolateral and apical staining of serous acini (Fig. 6C) and mucus acini (Fig. 6D), suggesting that tight junction integrity was affected likely due to excessive expression of inflammatory cytokines^37^,^45^,^46^. Interestingly, we observed significant loss of ZO-1 in the apical region of both serous acini (Fig. 6C as indicated in white arrows) and mucus acini (Fig. 6D as indicated in white arrows) where most of the ZO-1 staining was in the basolateral (Fig. 6C,D) of *ALX/FPR2*^−/−^ mice treated with LPS. Our results are consistent with previous studies that suggested that inflammatory cytokines cause loss of tight junction protein ZO-1 and salivary gland dysfunction^23^.

## Conclusion

**Figure 7:**
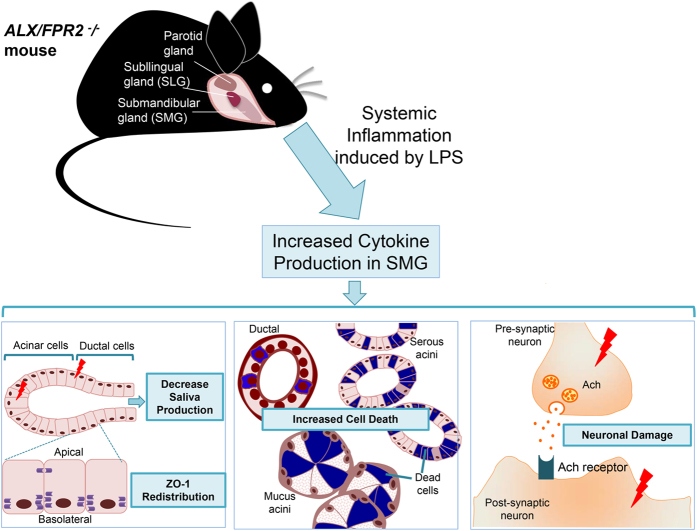
Protective role of *ALX/FPR2*^−/−^ in SMG. The diagram represents the effects of ALX/FPR2 in regulating LPS-mediated SMG acute inflammation. As indicated, lack of ALX/FPR2’s protection in SMG will cause hyposalivation, increase cell death and neuronal damage when stimulated with LPS. The schematic diagram was drawn by authors Ching-Shuen Wang and Yinshen Wee.

The acute inflammatory response is a critical mechanism for host defense^53^,^54^. Production of pro-inflammatory cytokines such as TNF-α, IL-6 and IFN-γ by local epithelial cells during acute inflammation lead to immune cell recruitment (*e.g*. macrophages and lymphocytes) and promotion of healing^19^,^55^. RvD1 is a potent lipid mediator that promotes tissue resolution by activating ALX/FPR2 leading to recruitment of neutrophils to the site of inflammation. Additionally, activation of this receptor improves tissue repair by stimulating different macrophage populations to clear cellular debris^53^,^56^,^57^. Our studies indicate that unresolved acute inflammation due to the absence of ALX/FPR2 alters leukocyte migration and cell-cell communication between phagocytes. Together, these events lead to chronic inflammation, scar tissue formation, pathological apoptosis as well as autoimmunity^11^,^12^,^26^,^8^,^29^,^30^,^35^. Salivary gland infection is usually accompanied with pain, swelling and decreased salivary flow^2^,^3^,^58^. Overall, the *ALX/FPR2*^−/−^ mouse model (Fig. 7) strongly suggested that xerostomia induced by LPS-triggered systematic infection is dramatically enhanced by the loss of ALX/FPR2 receptors.

## Materials and Methods

### Experimental animals

Wild type C57BL/6 mice were purchased from The Jackson Laboratory (Bar Harbor, Maine), *ALX/FPR2*^−/−^ mice were a generous gift from Dr. Mauro Perretti (William Harvey Research Institute, Barts and The London School of Medicine, United Kingdom). Groups of male and female mice at 8 weeks of age were genotyped as described previously^23^ (Supplementary Fig. 1) and treated via intraperitoneal (*i.p*) injection as follows: 1) C57BL/6 were injected with PBS (10 μl/g), 2) C57BL/6 were injected with LPS (Sigma, 10 mg/kg), 3) *ALX/FPR2*^−/−^ were injected with PBS (10 μl/g), 4) *ALX/FPR2*^−/−^ were injected with LPS injection (10 mg/kg). Twenty-four hours after PBS or LPS injection, mice were anesthetized with 100 mg/kg ketamine and 5 mg/kg xylazine and euthanized by abdominal exsanguination and the SMG removed for preparation of tissue sections, cell suspensions, RNA and protein. The animal protocol (14-006007) was reviewed and approved by the Institutional Animal Care and Use Committee (IACUC) at University of Utah, according to the animal welfare act of the United States (7 U.S.C. 2131 et. seq.). All mouse experiments were carried out at the animal care facility of the University of Utah, Colorow 383 building, in accordance with approved guidelines.

### Histological studies

SMG were immersed in 10% neutral formalin at room temperature (RT) for at least 24 h. SMG were then dehydrated in serial ethanol solutions (50%, 70% and 100% for 2 h each), embedded in paraffin wax and 7 μm sections obtained. Routine H&E (Hematoxylin and eosin) staining was performed and images were acquired by light microscopy (Leica Microsystems DMI6000B Inverted Microscope). The percentage of leukocytes infiltration was determined by calculating infiltrated area/total area of SMG samples from each group. Statistical results were analyzed and plotted using Prism software (GraphPad Software Inc.).

### FACs (Fluorescence-Activated Cell Sorting)

Mice were euthanized and fresh SMG were collected and single cells obtained by homogenization using a 100 μm cell strainer (Fisher Scientific). Briefly, the plunger end of a syringe was used to mash the SMG through the cell strainer into a 50 ml tube, followed by washing the cell strainer two times with 5 ml of 0.5% bovine serum albumin (BSA) in PBS. Cells were then centrifuged at 150 × g and washed twice with 0.5% BSA in PBS. Then, cells were split into two groups and labeled with the following fluorescent-conjugated primary antibodies (all from Biolegends): group A) APC anti-mouse F4/80, PerCP/Cy5.5 anti-mouse CD11b and PE/Cy7 anti-mouse CD45. Group B) PerCP/Cy5.5 anti-mouse B220, PE anti-mouse CD4, APC anti-mouse CD3ɛ, PE/Cy7 anti-mouse CD45 with Fc blocker at 1:400 dilutions using 0.5% BSA in PBS at RT for 30 min. Labeled cells were then washed with 0.5% BSA in PBS three times and then resuspended in 0.5 ml of 0.5% BSA in PBS and kept on ice until analysis. Finally, cells were probed with FACs (FACSCanto, BD Bioscience) and DAPI (4′,6-diamidino-2-phenylindole, 1 μg/ml) was added for sorting of dead cells. Results were analyzed using the FlowJo software (Tree Star).

### Quantitative Polymerase Chain Reaction (qPCR)

Fresh dissected mouse SMG was immersed in RNA stabilization solution (Invitrogen) at 4 °C overnight. Next day, the RNA stabilization solution was removed and total RNAs were extracted using the RNAeasy Kit (QIAGEN) according to manufacturer’s instructions. Total RNAs were reverse transcribed into cDNAs with iScript Reverse Transcription Kit according to manufacturer’s instructions (Bio-Rad). Finally, total cDNAs were diluted at 1:50 ratios and used as templates for qPCR. Briefly, the reactions were carried out by adding the following reagents: 2.5 μl of each primer^59^,^60^ (stock 10 μm, see Supplementary Table 1), 5 μl of 1:50 cDNA dilutions and 10 μl of 2× SYBR Green master mixes (Bio-Rad). PCR conditions were performed on 96 well plates at the following temperature cycles: step 1: 95 °C for 5 min; step 2: 95 °C for 30 s, 60 °C for 30 s and 72 °C for 35 s for 35 more cycles; step 3: 72 °C for 5 min; and add melting curve detection at final step to verify the specificity of amplicons. Relative fold changes of gene expression were normalized using β-actin, and results (see Supplementary Table 2 and 3) were plotted and analyzed using Prism software (GraphPad Software Inc.).

### Western blot analyses

Total proteins from freshly dissected mouse SMG were obtained using RIPA buffer containing a cocktail of protease inhibitors (Sigma) and dissociated with a Fisher Scientific Sonic Dismembrator (model FB-120; microtip; output level, 5; duty cycle, 50%; Thermo Fisher Scientific). Samples were centrifuged at 135,000 r.p.m. for 10 min to remove cellular debris. Protein concentration was determined by using the BCA protein determination Kit (Pierce). Then, samples were denatured by boiling in SDS sample buffer (Bio-Rad) 7 min. Fifty μg of total protein was loaded in each lane of a gradient 4%–15% SDS-PAGE gel (Bio-Rad) and then the electrophoresed samples were transferred to a nitrocellulose membrane (Bio-Rad). Subsequently, membranes were blocked for 1 h at RT using 1% BSA in Tris-buffered saline [0.137 M NaCl, 0.025 M Tris (hydroxymethyl)-aminomethane, pH 7.4] containing 0.1% Tween-20 (TBST) and immunoblotted overnight at 4 °C with the following primary antibodies in TBST containing 1% BSA: rabbit-anti-β-actin (Abcam; Cambridge, MA) 1:2500 dilutions, rabbit-anti-M3R (Novous) 1:1000 dilutions and rabbit anti-AQP5 (Abcam) 1:1000 dilutions. After incubation, membranes were washed three times for 5 min each with TBST and incubated with peroxidase-linked goat-anti-rabbit IgG antibody (Cell Signaling Technology) diluted 1:5000 in TBST containing 1% BSA at 4 °C overnight. The membranes were washed three times for 5 min each with TBST and treated with a Bio-Rad Clarity™ detection reagent (Bio-Rad). The protein bands were visualized using Bio-Rad ChemiDoc™ MP imager and band intensities were quantified using Image Lab 4.1 software (Bio-Rad). β-actin was used for protein normalization, and the relative protein expression level was presented as the ratio of M3R or AQP5 to actin. Western blots are shown in Supplementary Fig. 4 (male) and Fig. 5 (females). Statistical results were analyzed and plotted using Prism software (GraphPad Software Inc.).

### Saliva Collection

Mice were anesthetized with ketamine/xylaxine (100 mg/kg ketamine and 5 mg/kg xylazine), and injected with pilocarpine-HCl/PBS (Sigma) at 10 mg/kg via *i.p* to stimulate saliva secretion. Then, saliva was collected using a 200 μl pipette and placed immediately on ice in the presence of a protease inhibitor cocktail (Sigma). Both total weight and volume of saliva were measured by microbalance (Mettler Toledo) and pipettes (Eppendorf AG), respectively. Statistical results were analyzed and plotted by Prism (GraphPad Software Inc.).

### Immunofluorescence

Seven μm thick paraffin embedded mouse SMG sections from each group were deparaffinized with xylene and rehydrated with serial ethanol solutions (100%, 70% and 50%). Sections were rinsed with distilled water three times, and then incubated in sodium citrate buffer (10 mM sodium citrate, 0.05% Tween 20, pH 6.0) at 95 °C for 30 min. Sections were then washed with distilled water and permeabilized with 0.1% Triton X-100/PBS at RT for 45 min. Sections were then blocked in 5% rabbit serum in PBS for 1 h at RT, and incubated at 4 °C with anti-mouse β-III tubulin 1:250 dilutions (Abcam) and anti-rabbit ZO-1 1:250 dilutions in 5% goat serum overnight. Samples were washed three times for 5 min with PBS. Then, they were incubated for 1 h with anti-rabbit Alexa Fluor 488 secondary antibody 1:500 dilutions (Invitrogen) and anti-mouse Alexa Fluor 568 secondary antibody 1:500 dilutions in 5% goat serum at RT. Sections were then washed three times with PBS, for 5 min each. Subsequently, tissue sections were counter-stained with TO-PRO-3 Iodide (Invitrogen) at RT for 15 min at 1:1000 dilutions, then washed 3 times with PBS, for 5 min each. Finally, specimens were analyzed by fluorescence microscopy and analyzed using a confocal Zeiss LSM 700 microscope at 10× magnifications. A total depth of 7 μm was acquired for each sample, and total projection was visualized in the xy planes.

### TUNEL analysis

Seven μm thick paraffin embedded mouse SMG sections from each group were deparaffinized with xylene and rehydrated with serial ethanol solutions (100%, 70% and 50%). Sections were rinsed with distilled water three times before use and treated with proteinase K (Sigma, 10 ng/ml) at RT for 20 min, and then permeabilized with 0.25% Triton X-100/PBS at RT for 25 min. TUNEL (Terminal deoxynucleotidyl transferase dUTP nick end labeling) reaction was carried out according to manufacturer’s instructions (Invitrogen) in the dark at all times. Slides were then mounted with gold prolong anti-fade mounting medium (Invitrogen), and fluorescent signals were captured by fluorescence microscopy (Leica Microsystems DMI6000B Inverted Microscope). Percentage of apoptotic cells were quantified by calculating TUNEL-positive green cells from total cells stained with DAPI Nuclear Counterstains (ThermoFisher Scientific). At least 20 images were analyzed of each group, and statistical results were plotted using Prism Software (GraphPad Software Inc.).

### Statistical Analyses

Data are mean ± SD of the results from three or more experiments. *P-*values less than 0.05 were calculated from a two-tailed *t*-test or a two-way ANOVA with Prism (GraphPad Software Inc.) and taken to represent significant differences.

## Additional Information

**How to cite this article**: Wang, C.-S. *et al*. ALX/FPR2 Modulates Anti-Inflammatory Responses in Mouse Submandibular Gland. *Sci. Rep.* **6**, 24244; doi: 10.1038/srep24244 (2016).

## Supplementary Material

Supplementary Information
