# Supplementary material for: ALX/FPR2 Modulates Anti-Inflammatory Responses in Mouse Submandibular Gland
Source: Sci Rep. 2016 Apr 11;6:24244. doi: 10.1038/srep24244 (PMC4827125; doi:10.1038/srep24244)
Supplement: Supplementary Information [file srep24244-s1.pdf]

Supplementary Figures

ALX/FPR2 Modulates Anti-Inflammatory Responses in Mouse Submandibular Gland

Ching-Shuen Wang[1], Yinshen Wee[2], Chieh-Hsiang Yang[3], James E. Melvin[4] and Olga J. Baker*[1]

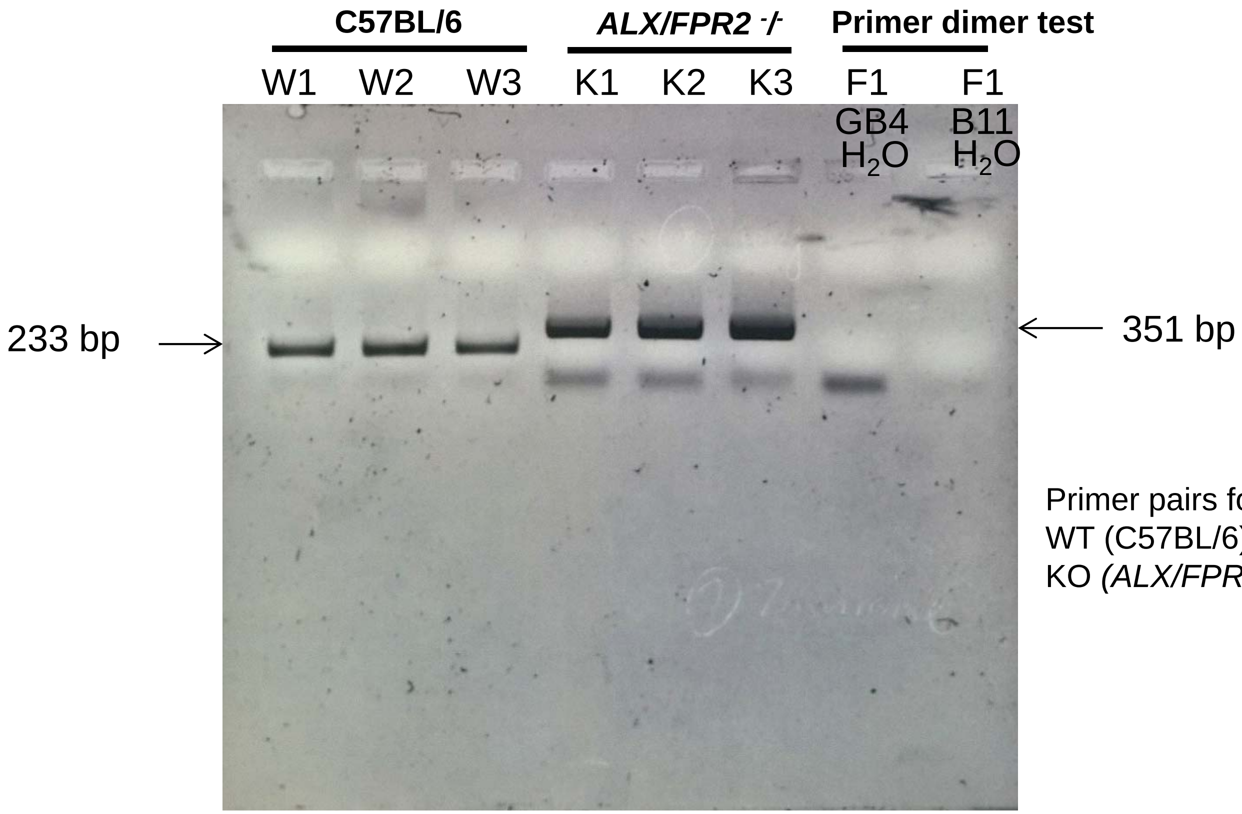

Primer pairs for WT and KO mice.
WT (C57BL/6) : F1+B11 primer set, 233 bp
KO *(ALX/FPR2 $^{-/-}$)*: F1+GB4 primer set, 351 bp

Supplementary Fig 1. ALX/FPR2 $^{-/-}$ genotyping. The mice obtained from Dr. Perretti's group (William Harvey Research Institute, Barts and The London School of Medicine, London EC1M 6BQ, United Kingdom) were genotyped and compared to wildtype C57BL/6 mice. PCR amplified samples were separated on 2% agarose gel and post-stained with SYBR fluorescent dye to visualize DNA fragments. Correct size of band from ALX/FPR2 $^{-/-}$ genome sample appeared in 351 bp whereas C57BL/6 mice showed 233 bp bands.

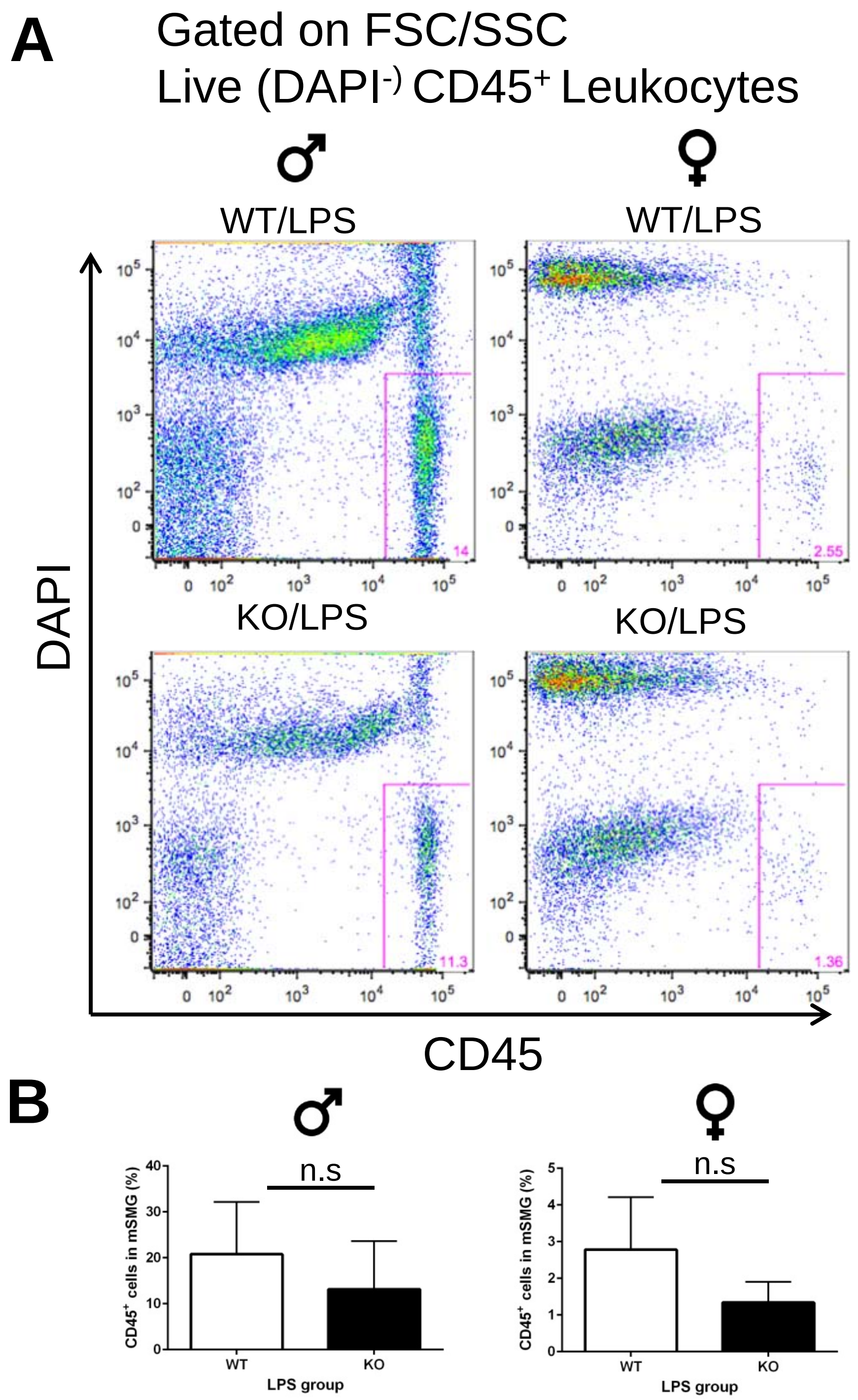

Supplementary Fig. 2 *ALX/FPR2 -/-* mice treated with LPS showed no statistical significance of SMG leukocyte infiltration. A) Representative FACs analysis of CD45+ cells in male or female SMG from LPS treatment groups. CD45+ cells are shown in the pink box. B) Statistical analysis of CD45+ cells. Abbreviations: *WT/LPS* wildtype C57BL/6 mice injected with LPS, and *KO/LPS ALX/FPR2 -/-* mice injected with LPS. N=6 mice were used were used for each experimental group. n.s., no significance.

	Gene	F-primer (5’-3’)	R-primer (5’-3’)
**Control**	*β-actin*	GTAACAATGCCATGTTCAAT	CTCCATCGTGGGCCGCTCTAG
**Pro-inflammatory**	*IFN-γ*	GGCCATCAGCAACAACATAAGCGT	TGGGTTGTTGACCTCAAACTTGGC
	IFN-β	AACCTCACCTACAGGGCGGACTTCA	TCCCACGTCAATCTTTCCTCTTGCTTT
	IL-6	TCCAGTTGCCTTCTTGGGAC	GTACTCCAGAAGACCAGAGG
	TNF-α	CTGTAGCCCACGTCGTAGC	TTGAGATCCATGCCGTTG
**Genotyping primers**	*WT*	TGAGTGTCATGTCAGAAGGAGCC	CGGAATCCAGCTACCCAAATC
**Genotyping primers**	*ALX/FPR2* $^{-/-}$	TGAGTGTCATGTCAGAAGGAGCC	ATAACCTTCGGGCATGGCACTC

Supplementary Table 1. Primer sequences used in this study. Primer sequences were adapted from previous studies[25-26].

♂

	WT/PBS						KO/PBS						WT/LPS						KO/LPS					
	M1	M2	M3	M4	M5	M6	M1	M2	M3	M4	M5	M6	M1	M2	M3	M4	M5	M6	M1	M2	M3	M4	M5	M6
b-actin Ct	23.66979	23.53050	24.42564	22.38942	19.43891	23.64630	24.40465	24.64819	24.22980	24.57671	19.42776	24.52744	19.42776	24.62764	24.36379	24.89831	19.35003	24.46642	24.19831	24.01000	23.75499	24.99412	18.15513	24.73538
IL-6 Ct	39.22131	37.94329	39.50000	37.72207	34.76908	37.89523	38.30089	39.18294	39.93307	40.00000	37.08702	39.53632	36.13853	33.11316	33.16322	33.31941	27.17590	33.52427	29.99673	30.41181	29.81758	31.53507	32.44557	30.53632
dCT IL-6	-15.55152	-14.41279	-15.07436	-15.33265	-15.33016	-14.24893	-13.89624	-14.53475	-15.70327	-15.42329	-17.65926	-15.00888	-16.71077	-8.48552	-8.79943	-8.42110	-7.82587	-9.05785	-5.79842	-6.40181	-6.06260	-6.54095	-14.29044	-5.80094
fold change	0.00002	0.00005	0.00003	0.00002	0.00002	0.00005	0.00007	0.00004	0.00002	0.00002	0.00000	0.00003	0.00001	0.00279	0.00224	0.00292	0.00441	0.00188	0.01797	0.01183	0.01496	0.01074	0.00005	0.01794
x2500 fold change	0.05206	0.11462	0.07246	0.06058	0.06069	0.12841	0.16397	0.10533	0.04686	0.05689	0.01208	0.07583	0.02331	6.97499	5.61109	7.29351	11.01837	4.69089	44.92022	29.56672	37.40390	26.84840	0.12476	44.84180

	M1	M2	M3	M4	M5	M6	M1	M2	M3	M4	M5	M6	M1	M2	M3	M4	M5	M6	M1	M2	M3	M4	M5	M6
b-actin Ct	23.66979	23.53050	24.42564	22.38942	19.43891	22.52352	24.40465	24.64819	24.22980	24.57671	19.42776	24.52522	24.83056	24.62764	24.36379	24.89831	19.35003	24.25563	24.19831	24.01000	23.75499	24.99412	18.15513	23.52356
TNF-a Ct	33.78000	33.77000	34.87000	33.14748	29.81409	33.63211	35.20000	35.33336	34.33952	35.25600	30.36000	35.26747	31.78000	31.05862	31.05000	32.20000	26.47300	31.63463	29.64000	30.01204	28.35476	30.41000	23.65000	28.53232
dCT TNF-a	-10.11021	-10.23950	-10.44436	-10.75806	-10.37518	-11.10859	-10.79535	-10.68517	-10.10972	-10.67929	-10.93224	-10.74225	-6.94944	-6.43099	-6.68621	-7.30169	-7.12297	-7.37900	-5.44169	-6.00204	-4.59978	-5.41588	-5.49487	-5.00876
fold change	0.00090	0.00083	0.00072	0.00058	0.00075	0.00045	0.00056	0.00061	0.00091	0.00061	0.00051	0.00058	0.00809	0.01159	0.00971	0.00634	0.00717	0.00601	0.02301	0.01560	0.04124	0.02342	0.02218	0.03106
x2500 fold change	2.26185	2.06797	1.79422	1.44358	1.88235	1.13219	1.40675	1.51839	2.26262	1.52459	1.27940	1.45949	20.22780	28.97476	24.27668	15.84577	17.93543	15.01895	57.52137	39.00726	103.10257	58.55962	55.43961	77.65206

	M1	M2	M3	M4	M5	M6	M1	M2	M3	M4	M5	M6	M1	M2	M3	M4	M5	M6	M1	M2	M3	M4	M5	M6
b-actin Ct	23.66979	23.53050	24.42564	22.38942	19.43891	23.52353	24.40465	24.64819	24.22980	24.57671	19.42776	24.21536	24.83056	24.62764	24.36379	24.89831	19.35003	24.23563	24.19831	24.01000	23.75499	24.99412	18.15513	24.65282
IFN-g Ct	36.67820	35.78829	37.05468	34.15172	34.57646	35.25373	37.41404	36.87200	36.99565	36.82370	35.51909	36.52632	36.65081	36.34174	36.23551	36.27686	32.14717	36.53634	35.18321	35.14111	34.99143	35.52647	32.23647	35.73636
dCT IFN-g	-13.00841	-12.25779	-12.62904	-11.76230	-15.13754	-11.73020	-13.00938	-12.22382	-12.76584	-12.24699	-16.09133	-12.31096	-11.82025	-11.71410	-11.87172	-11.37855	-12.79714	-12.30071	-10.98489	-11.13110	-11.23645	-10.53235	-14.08134	-11.08354
fold change	0.00012	0.00020	0.00016	0.00029	0.00003	0.00029	0.00012	0.00021	0.00014	0.00021	0.00001	0.00020	0.00028	0.00030	0.00027	0.00038	0.00014	0.00020	0.00049	0.00045	0.00041	0.00068	0.00006	0.00046
x2500 fold change	0.30340	0.51048	0.39466	0.71967	0.06936	0.73586	0.30320	0.52264	0.35895	0.51431	0.03581	0.49201	0.69134	0.74412	0.66711	0.93898	0.35125	0.49552	1.23355	1.11466	1.03617	1.68806	0.14422	1.15203

	M1	M2	M3	M4	M5	M6	M1	M2	M3	M4	M5	M6	M1	M2	M3	M4	M5	M6	M1	M2	M3	M4	M5	M6
b-actin Ct	23.66979	23.53050	24.42564	22.38942	19.43891	23.56363	24.40465	24.64819	24.22980	24.57671	19.42776	24.63673	24.83056	24.62764	24.36379	24.89831	19.35003	23.64754	24.19831	24.01000	23.75499	24.99412	18.15513	23.59365
IFN-b Ct	35.18576	35.14238	36.54638	35.57736	32.25479	35.72889	34.06529	34.28031	34.31339	36.82901	31.21475	34.72758	34.54582	35.78017	33.89343	34.30374	29.14868	35.56363	32.73764	32.23544	33.06797	33.49625	26.85320	35.53286
dCT IFN-b	-11.51597	-11.61188	-12.12074	-13.18794	-12.81587	-12.16526	-9.66063	-9.63212	-10.08359	-12.25230	-11.78699	-10.09085	-9.71526	-11.15254	-9.52964	-9.40543	-9.79865	-11.91609	-8.53933	-8.22544	-9.31298	-8.50212	-8.69807	-11.04278
fold change	0.00034	0.00032	0.00022	0.00011	0.00014	0.00022	0.00124	0.00126	0.00092	0.00020	0.00028	0.00092	0.00119	0.00044	0.00135	0.00147	0.00112	0.00026	0.00269	0.00334	0.00157	0.00276	0.00241	0.00047
x2500 fold change	0.85367	0.79876	0.56135	0.26790	0.34672	0.54429	3.08887	3.15052	2.30398	0.51243	0.70746	2.29241	2.97409	1.09822	3.38246	3.68658	2.80706	0.64690	6.71965	8.35289	3.93056	6.89518	6.01951	1.18504

	M1	M2	M3	M4	M5	M6	M1	M2	M3	M4	M5	M6	M1	M2	M3	M4	M5	M6	M1	M2	M3	M4	M5	M6
b-actin Ct	23.66979	23.53050	24.00000	24.40000	22.60000	22.72463	24.40465	23.80000	24.22980	24.57671	21.40000	22.63174	24.83056	24.10000	25.30000	24.89831	23.00000	22.35223	24.19831	24.01000	23.75499	24.99412	18.15513	20.23163
mM3R Ct	28.64566	28.55612	28.01616	29.50371	27.64050	28.27636	28.41423	28.25616	28.36849	29.51068	26.44260	30.47864	29.63354	28.39464	30.04525	29.75692	28.48705	28.63184	30.82218	29.68384	29.96986	30.11149	26.61003	29.55232
dCT mM3R	-4.97587	-5.02562	-4.01616	-5.10371	-5.04050	-5.55173	-4.00958	-4.45616	-4.13868	-4.93397	-5.04260	-7.71390	-4.80299	-4.29464	-4.74525	-4.85860	-5.48705	-6.42901	-6.62387	-5.67384	-6.21487	-5.11737	-8.45490	-9.30639
fold change	0.03178	0.03070	0.06180	0.02908	0.03038	0.07207	0.06209	0.04556	0.05677	0.03271	0.03034	0.00523	0.03582	0.05095	0.03729	0.03447	0.02230	0.02188	0.01014	0.01959	0.01346	0.02881	0.00285	0.00319
x2500 fold change	79.44291	76.74964	154.50972	72.70576	75.96215	65.17651	155.21592	113.89407	141.92924	81.78384	75.85186	61.33578	89.55628	127.38648	93.21314	86.16967	55.74087	52.19361	25.34883	48.97163	33.65714	72.02090	7.12462	33.96418

	M1	M2	M3	M4	M5	M6	M1	M2	M3	M4	M5	M6	M1	M2	M3	M4	M5	M6	M1	M2	M3	M4	M5	M6
b-actin Ct	20.59070	20.75659	21.43139	21.43847	21.43146	22.53686	21.59942	21.49779	21.23601	22.38210	21.44247	21.11563	21.65137	21.63251	21.43525	21.48508	21.25798	24.12546	22.12661	20.96193	20.96546	22.23075	19.93072	20.41432
AQUP5 Ct	24.96775	24.92290	24.43113	25.29062	25.21937	25.61164	25.46463	25.21434	24.90568	26.05450	24.87839	25.63724	26.73622	25.73209	27.45847	27.06618	26.54327	27.62582	27.10076	26.17589	26.14775	26.58305	25.00333	25.65644
dCt	-4.37705	-4.16631	-2.99974	-3.85215	-3.78791	-3.03338	-3.86521	-3.71656	-3.66967	-3.67241	-3.43592	-3.963071	-5.08485	-4.09958	-6.02323	-5.58110	-5.28528	-3.31636	-4.97415	-5.21396	-5.18229	-4.35230	-5.07261	-4.93112
fold	0.04813	0.05569	0.12502	0.06924	0.07240	0.23410	0.06862	0.07607	0.07858	0.07843	0.09240	0.042423	0.02947	0.05833	0.01538	0.02089	0.02564	0.26445	0.03181	0.02694	0.02754	0.04896	0.02972	0.02212
x2500 fold change	120.31426	139.23715	312.55620	173.11168	180.99513	115.25967	171.55213	190.17126	196.45316	196.08112	231.00763	260.56375	73.66266	145.82876	38.43869	52.22294	64.10780	61.12540	79.53733	67.35679	68.85185	122.39637	74.29030	78.54884

Supplementary Table 2. qPCR raw data of all male mice. qPCR results were obtained from Ct (cycle numbers) values, and normalized to actin to get ∆Ct values. The difference of fold changes between sample and actin are 2 to the power of ∆Ct Values. Statistical analysis were performed by using Prism. Abbreviations: *WT PBS* wildtype C57BL/6 mice injected with PBS, *KO PBS ALX/FPR2* $^{-/-}$ mice injected with PBS, *WT LPS* wildtype C57BL/6 mice injected with LPS, and *KO LPS ALX/FPR2* $^{-/-}$ mice injected with LPS. N=6 mice were used for each group of study.

♀

	WT/PBS						KO/PBS						WT/LPS						KO/LPS					
	**M1**	**M2**	**M3**	**M4**	**M5**	**M6**	**M1**	**M2**	**M3**	**M4**	**M5**	**M6**	**M1**	**M2**	**M3**	**M4**	**M5**	**M6**	**M1**	**M2**	**M3**	**M4**	**M5**	**M6**
**Actin Ct**	21.11994	22.03466	22.42603	22.59073	23.76311	22.18383	21.21848	21.47138	22.02538	22.14545	23.30009	22.17362	22.43682	22.65257	24.39361	22.91454	24.24578	22.63326	20.99469	21.25240	20.98901	24.58928	24.00213	24.64743
**IL-6 Ct**	36.25179	37.56349	36.68354	37.33446	38.00000	37.67233	29.40000	29.70000	30.26690	29.25400	30.40000	29.56364	38.44226	36.34820	38.00000	35.52189	37.37286	36.52785	27.40801	27.26005	27.74700	30.19540	29.65181	29.52353
**dCt**	-15.13184	-15.52882	-14.25751	-14.74373	-14.23689	-15.48850	-8.18152	-8.22862	-8.24151	-7.10855	-7.09991	-7.39002	-16.00544	-13.69563	-13.60639	-12.60735	-13.12708	-13.89459	-6.41331	-6.00764	-6.75799	-5.60612	-5.64968	-4.87610
**fold**	0.00003	0.00002	0.00005	0.00004	0.00005	0.00002	0.00344	0.00333	0.00330	0.00725	0.00729	0.00596	0.00002	0.00008	0.00008	0.00016	0.00011	0.00007	0.01173	0.01554	0.00924	0.02053	0.01992	0.03405
**x2500 fold change**	0.06963	0.05288	0.12764	0.09112	0.12948	0.05438	8.61110	8.33450	8.26032	18.11564	18.22447	14.90467	0.03800	0.18843	0.20045	0.40063	0.27944	0.16415	29.33190	38.85607	23.09848	51.32502	49.79849	85.13098
	**M1**	**M2**	**M3**	**M4**	**M5**	**M6**	**M1**	**M2**	**M3**	**M4**	**M5**	**M6**	**M1**	**M2**	**M3**	**M4**	**M5**	**M6**	**M1**	**M2**	**M3**	**M4**	**M5**	**M6**
**Actin Ct**	21.11994	22.03466	22.42603	22.59073	23.76311	22.52752	21.21848	21.47138	22.02538	22.14545	23.30009	22.54263	22.43682	22.65257	24.39361	22.91454	24.24578	22.53674	20.99469	21.25240	20.98901	24.58928	24.00213	21.23536
**TNF-a Ct**	31.15416	31.94559	33.66810	33.44576	35.66843	33.62675	29.77675	29.39581	30.85548	29.67235	32.04670	29.56363	32.15334	32.48228	35.02902	32.65884	34.13956	32.53646	28.50000	27.37340	27.70000	31.25000	29.50879	27.52523
**dCT**	-10.03421	-9.91093	-11.24206	-10.85503	-11.90532	-11.09923	-8.55827	-7.92443	-8.83010	-7.52690	-8.74660	-7.02100	-9.71652	-9.82971	-10.63541	-9.74430	-9.89377	-9.99972	-7.50531	-6.12100	-6.71099	-6.66072	-5.50666	-6.28987
**fold**	0.00095	0.00104	0.00041	0.00054	0.00026	0.00046	0.00265	0.00412	0.00220	0.00542	0.00233	0.00770	0.00119	0.00110	0.00063	0.00117	0.00105	0.00098	0.00550	0.01437	0.00955	0.00988	0.02200	0.01278
**x2500 fold change**	2.38419	2.59689	1.03215	1.34975	0.65175	1.13956	6.63201	10.29081	5.49306	13.55553	5.82036	19.24901	2.97149	2.74728	1.57167	2.91483	2.62795	2.44188	13.75997	35.92002	23.86337	24.70944	54.98831	31.95218
	**M1**	**M2**	**M3**	**M4**	**M5**	**M6**	**M1**	**M2**	**M3**	**M4**	**M5**	**M6**	**M1**	**M2**	**M3**	**M4**	**M5**	**M6**	**M1**	**M2**	**M3**	**M4**	**M5**	**M6**
**Actin Ct**	21.11994	22.03466	22.42603	22.59073	23.76311	22.53563	21.21848	21.47138	22.02538	22.14545	23.30009	22.15364	22.43682	22.65257	24.39361	22.91454	24.24578	24.52632	20.99469	21.25240	20.98901	24.58928	24.00213	24.52523
**IFN-g Ct**	33.79510	35.06519	35.84383	34.51335	36.13855	35.25252	32.33662	33.09566	33.75380	33.57363	34.57420	33.64642	37.30827	36.10551	38.16161	34.42880	36.30827	36.67352	31.23780	32.16475	31.21546	35.04274	34.29043	34.56353
**dCt**	-12.67515	-13.03053	-13.41780	-11.92262	-12.37544	-12.71689	-11.11814	-11.62428	-11.72842	-11.42818	-11.27411	-11.49278	-14.87146	-13.45294	-13.76800	-11.51426	-12.06249	-12.14720	-10.24311	-10.91235	-10.22645	-10.45346	-10.28830	-10.03830
**fold**	0.00015	0.00012	0.00009	0.00026	0.00019	0.00015	0.00045	0.00032	0.00029	0.00036	0.00040	0.00035	0.00003	0.00009	0.00007	0.00034	0.00023	0.00022	0.00083	0.00052	0.00083	0.00071	0.00080	0.00095
**x2500 fold change**	0.38224	0.29879	0.22844	0.64398	0.47050	0.37134	1.12473	0.79192	0.73677	0.90723	1.00947	0.86750	0.08340	0.22295	0.17921	0.85468	0.58448	0.55115	2.06280	1.29717	2.08676	1.78293	1.99919	2.37745
	**M1**	**M2**	**M3**	**M4**	**M5**	**M6**	**M1**	**M2**	**M3**	**M4**	**M5**	**M6**	**M1**	**M2**	**M3**	**M4**	**M5**	**M6**	**M1**	**M2**	**M3**	**M4**	**M5**	**M6**
**Actin Ct**	21.11994	22.03466	22.42603	22.59073	23.76311	22.53465	21.21848	21.47138	22.02538	22.14545	23.30009	22.41453	22.43682	22.65257	24.39361	22.91454	24.24578	22.56363	20.99469	21.25240	20.98901	24.58928	24.00213	20.69222
**IFN-b Ct**	38.04810	37.15932	38.00000	34.89720	38.19625	38.42353	33.09995	33.28000	32.81830	31.97637	33.23090	32.31654	38.00000	38.63812	39.00000	39.39284	37.52314	38.25378	31.36940	30.26880	30.47580	33.46789	33.46000	30.12647
**dCt**	-16.92816	-15.12466	-15.57397	-12.30647	-14.43314	-15.88888	-11.88147	-11.80862	-10.79292	-9.83092	-9.93081	-9.90201	-15.56318	-15.98555	-14.60639	-16.47831	-13.27736	-15.69015	-10.37471	-9.01640	-9.48679	-8.87861	-9.45787	-9.43425
**fold**	0.00001	0.00003	0.00002	0.00020	0.00005	0.00002	0.00027	0.00028	0.00056	0.00110	0.00102	0.00105	0.00002	0.00002	0.00004	0.00001	0.00010	0.00002	0.00075	0.00193	0.00139	0.00212	0.00142	0.00145
**x2500 fold change**	0.02005	0.06998	0.05125	0.49354	0.11301	0.04120	0.66262	0.69693	1.40912	2.74497	2.56135	2.61299	0.05164	0.03853	0.10023	0.02738	0.25180	0.04729	1.88296	4.82763	3.48444	5.31144	3.55498	3.61366
	**M1**	**M2**	**M3**	**M4**	**M5**	**M6**	**M1**	**M2**	**M3**	**M4**	**M5**	**M6**	**M1**	**M2**	**M3**	**M4**	**M5**	**M6**	**M1**	**M2**	**M3**	**M4**	**M5**	**M6**
**Actin Ct**	21.11994	22.03466	22.42603	22.59073	23.76311	22.75463	21.21848	21.47138	22.02538	22.14545	23.30009	22.63674	22.43682	22.65257	24.39361	22.91454	24.24578	22.35563	20.99469	21.25240	20.98901	24.58928	24.00213	20.23563
**M3R Ct**	27.64143	28.21794	28.69055	28.30192	29.51625	28.25636	29.00620	28.95062	30.12816	31.50151	31.13727	30.42164	28.26871	28.76931	29.77586	30.27990	31.11541	28.63464	29.01835	29.01205	28.59476	32.27216	32.12890	29.53632
**dCT**	-6.52148	-6.18328	-6.26451	-5.71119	-5.75314	-5.50173	-7.78772	-7.47924	-8.10277	-9.35606	-7.83718	-7.78490	-5.83189	-6.11674	-5.38225	-7.36536	-6.86963	-6.27901	-8.02366	-7.75964	-7.60575	-7.68288	-8.12677	-9.30069
**fold**	0.01089	0.01376	0.01301	0.01909	0.01854	0.02207	0.00453	0.00560	0.00364	0.00153	0.00437	0.00453	0.01756	0.01441	0.02398	0.00606	0.00855	0.01288	0.00384	0.00461	0.00513	0.00487	0.00358	0.00159
**x2500 fold change**	27.21310	34.40232	32.51871	47.72014	46.35245	55.17651	11.31366	14.01087	9.09415	3.81493	10.93234	11.33578	43.88993	36.02614	59.94057	15.16163	21.37845	32.19361	9.60679	11.53599	12.83458	12.16639	8.94411	3.96418
	**M1**	**M2**	**M3**	**M4**	**M5**	**M6**	**M1**	**M2**	**M3**	**M4**	**M5**	**M6**	**M1**	**M2**	**M3**	**M4**	**M5**	**M6**	**M1**	**M2**	**M3**	**M4**	**M5**	**M6**
**Actin Ct**	21.11994	22.03466	22.42603	22.59073	23.76311	22.63636	21.21848	21.47138	22.02538	22.14545	23.30009	21.67563	22.43682	22.65257	24.39361	22.91454	24.24578	24.63646	20.99469	21.25240	20.98901	24.58928	24.00213	20.75432
**AQUP5 Ct**	23.76809	23.77681	25.14180	24.65793	26.44512	25.67464	25.22546	25.33869	27.61021	27.00141	27.25855	25.63634	24.44014	24.66843	25.80563	26.12837	27.03178	27.64282	25.45215	25.21647	25.61390	28.70923	27.82834	25.69244
**dCT**	-2.64814	-1.74215	-2.71577	-2.06720	-2.68201	-3.01038	-4.00698	-3.86730	-5.58483	-4.85596	-3.95845	-3.96071	-2.00332	-2.01586	-1.41202	-3.21383	-2.78600	-3.00636	-4.45746	-3.96407	-4.62489	-4.11996	-3.82621	-4.93812
**fold**	0.15953	0.29892	0.15222	0.23862	0.15582	0.12410	0.06220	0.06852	0.02084	0.03453	0.06433	0.06423	0.24943	0.24727	0.37578	0.10778	0.14499	0.12445	0.04552	0.06408	0.04053	0.05751	0.07050	0.03262
**x2500 fold change**	398.81350	747.31107	380.55051	596.55452	389.56131	310.25967	155.49598	171.30346	52.08829	86.32779	160.81498	160.56375	623.56256	618.16626	939.46147	269.45094	362.46953	311.12540	113.79209	160.19077	101.32361	143.78374	176.25266	81.54884

Supplementary Table 3. qPCR raw data of all female mice. qPCR results were obtained from Ct (cycle numbers) values, and normalized to actin to get ΔCt values. The difference of fold changes between sample and actin are 2 to the power of ΔCt Values. Statistical analysis were performed by using Prism. Abbreviations: *WT PBS* wildtype C57BL/6 mice injected with PBS, *KO PBS ALX/FPR2 -/-* mice injected with PBS, *WT LPS* wildtype C57BL/6 mice injected with LPS, and *KO LPS ALX/FPR2 -/-* mice injected with LPS. N=6 mice were used for each group of study.

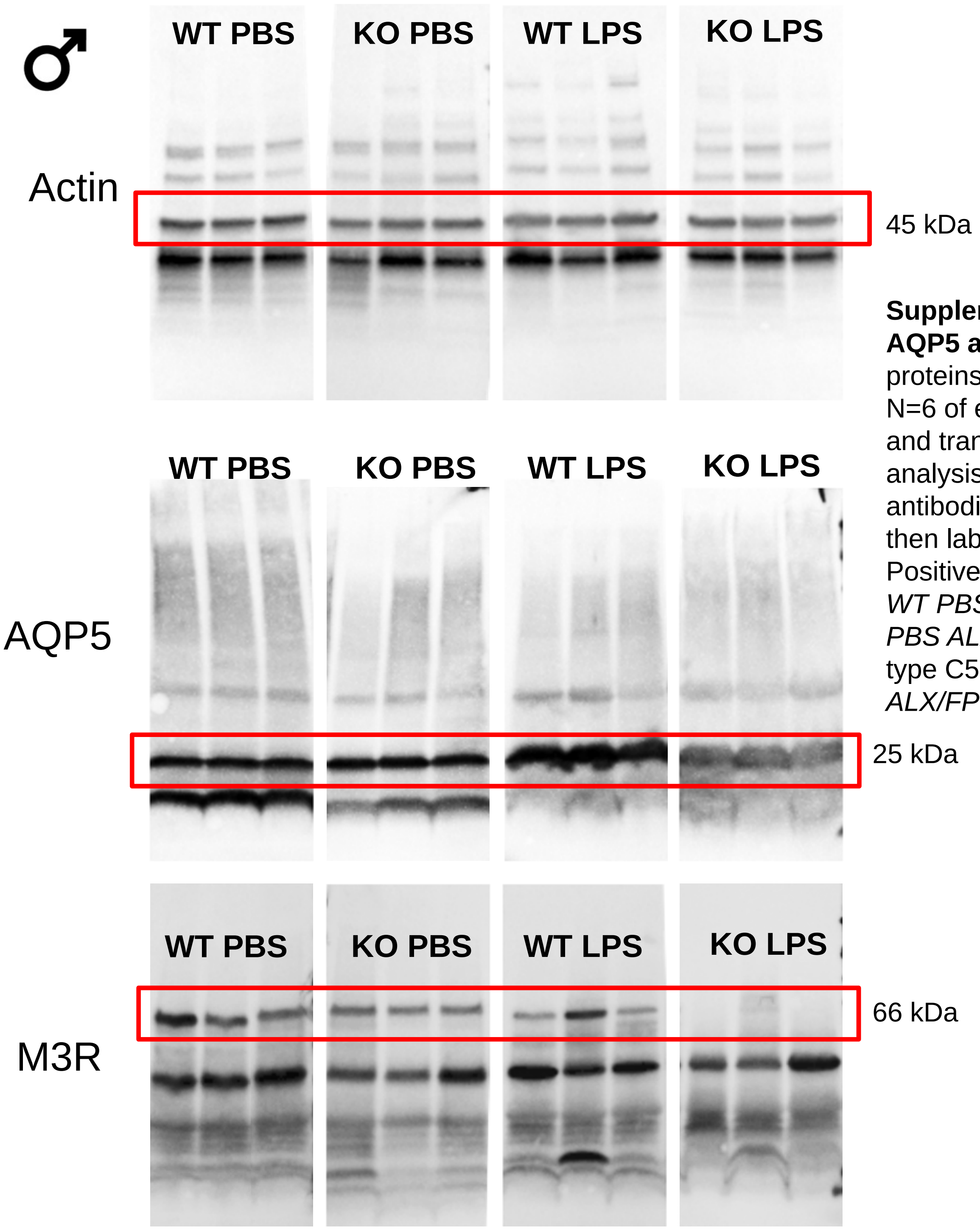

Supplementary Fig 4. Western blot analysis of actin, AQP5 and M3R from male groups. Same amount of total proteins from three different mice (representative blots from N=6 of each group) were subjected to a 4-15% SDS-PAGE, and transferred to nitrocellulose membrane for western blot analysis. Blots were stained with corresponding primary antibodies (actin, AQP5 and M3R at 1:1000 dilutions) and then labeled with anti-rabbit-HRP for band detection. Positive bands were highlighted in red boxes. Abbreviations: *WT PBS* wildtype C57BL/6 mice injected with PBS, *KO PBS ALX/FPR2* $^{-/-}$ mice injected with PBS, *WT LPS* wild type C57BL/6 mice injected with LPS, and *KO LPS ALX/FPR2* $^{-/-}$ mice injected with LPS.

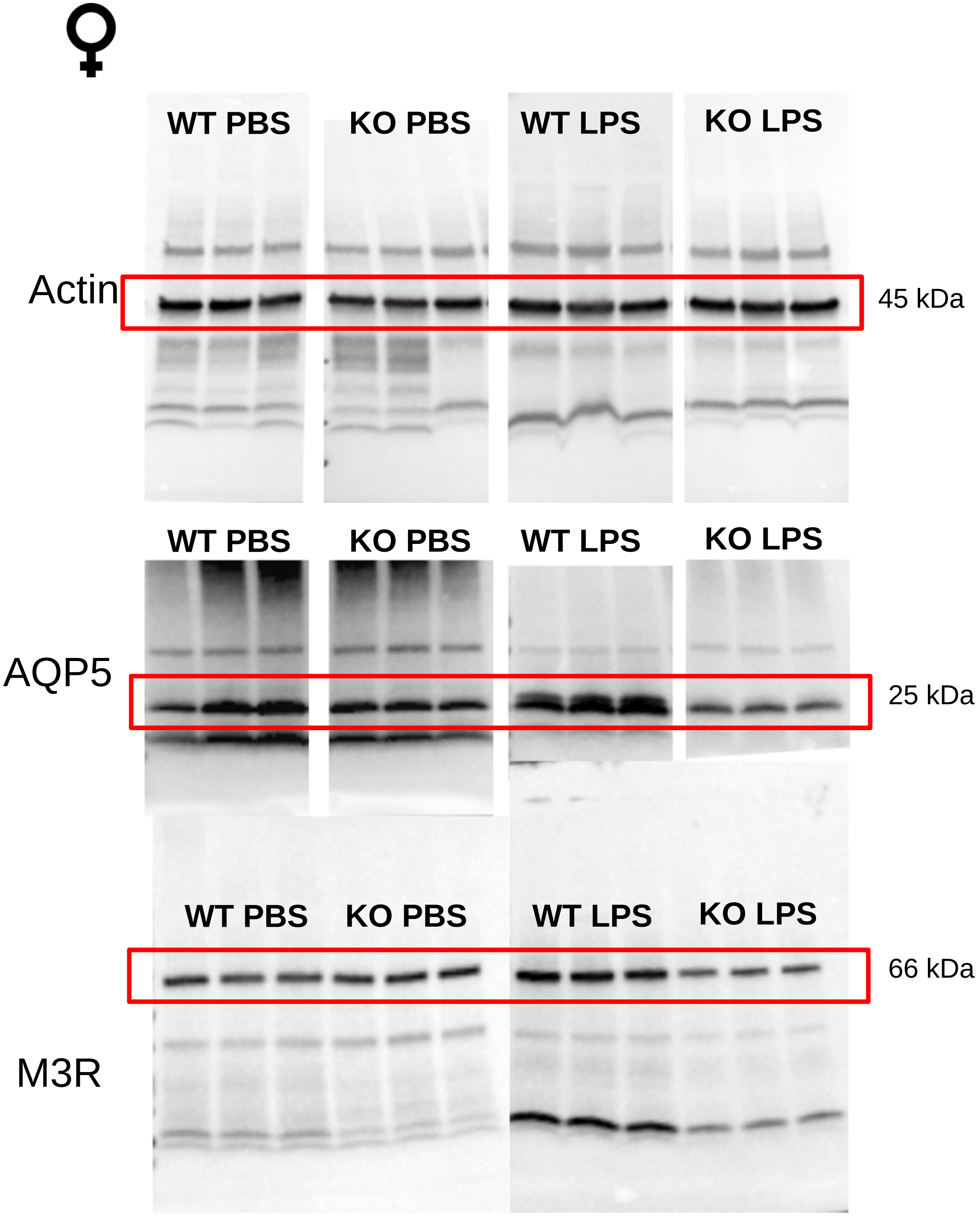

Supplementary Fig 5. Western blot analysis of actin, AQP5 and M3R from female groups. Same amount of total proteins from three different mice (representative blots from N=6 of each group) were subjected to a 4-15% SDS-PAGE, and transferred to nitrocellulose membrane for western blot analysis. Blots were stained with corresponding primary antibodies (actin, AQP5 and M3R at 1:1000 dilutions) and then labeled with anti-rabbit-HRP for band detection. Positive bands were highlighted in red boxes. Abbreviations: *WT PBS* wild type C57BL/6 mice injected with PBS, *KO PBS ALX/FPR2* $^{-/-}$ mice injected with PBS, *WT LPS* wildtype C57BL/6 mice injected with LPS, and *KO LPS ALX/FPR2* $^{-/-}$ mice injected with LPS.
